# Identification of a Minimal 3-Transcript Signature to Differentiate Viral from Bacterial Infection from Best Genome-Wide Host RNA Biomarkers: A Multi-Cohort Analysis

**DOI:** 10.3390/ijms22063148

**Published:** 2021-03-19

**Authors:** Alberto Gómez-Carballa, Ruth Barral-Arca, Miriam Cebey-López, Xabier Bello, Jacobo Pardo-Seco, Federico Martinón-Torres, Antonio Salas

**Affiliations:** 1 GenPoB Research Group, Instituto de Investigación Sanitaria (IDIS), Hospital Clínico Universitario de Santiago (SERGAS), 15706 Galicia, Spain; Alberto.Gomez.Carballa@sergas.es (A.G.-C.); barralarcaruth@gmail.com (R.B.-A.); Miriam.Cebey.Lopez@sergas.es (M.C.-L.); xbello@gmail.com (X.B.); Jacobo.Jose.Pardo.Seco@sergas.es (J.P.-S.); 2 Genetics, Vaccines and Infections Research Group (GENVIP), Instituto de Investigación Sanitaria de Santiago de Compostela, 15706 Galicia, Spain; federico.martinon.torres@sergas.es; 3 Translational Pediatrics and Infectious Diseases, Department of Pediatrics, Hospital Clínico Universitario de Santiago de Compostela, 15706 Galicia, Spain; 4 Unidade de Xenética, Instituto de Ciencias Forenses, Facultade de Medicina, Universidade de Santiago de Compostela, 15706 Galicia, Spain

**Keywords:** RNA, RNAseq, microarrays, transcriptome, transcriptomic biomarkers, RNA signature, multi-cohort, viral infection, bacterial infection, machine learning

## Abstract

The fight against the spread of antibiotic resistance is one of the most important challenges facing health systems worldwide. Given the limitations of current diagnostic methods, the development of fast and accurate tests for the diagnosis of viral and bacterial infections would improve patient management and treatment, as well as contribute to reducing antibiotic misuse in clinical settings. In this scenario, analysis of host transcriptomics constitutes a promising target to develop new diagnostic tests based on the host-specific response to infections. We carried out a multi-cohort meta-analysis of blood transcriptomic data available in public databases, including 11 different studies and 1209 samples from virus- (*n* = 695) and bacteria- (*n* = 514) infected patients. We applied a Parallel Regularized Regression Model Search (PReMS) on a set of previously reported genes that distinguished viral from bacterial infection to find a minimum gene expression bio-signature. This strategy allowed us to detect three genes, namely *BAFT*, *ISG15* and *DNMT1*, that clearly differentiate groups of infection with high accuracy (training set: area under the curve (AUC) 0.86 (sensitivity: 0.81; specificity: 0.87); testing set: AUC 0.87 (sensitivity: 0.82; specificity: 0.86)). *BAFT* and *ISG15* are involved in processes related to immune response, while *DNMT1* is related to the preservation of methylation patterns, and its expression is modulated by pathogen infections. We successfully tested this three-transcript signature in the 11 independent studies, demonstrating its high performance under different scenarios. The main advantage of this three-gene signature is the low number of genes needed to differentiate both groups of patient categories.

## 1. Introduction

According to the World Health Organization (WHO), infectious diseases are still among the major causes of child mortality and are responsible for many medical visits and hospitalizations around the globe [1]. Until recently, it was commonly considered that most severe infections were caused by bacterial pathogens but, during the last decade, increasing evidence shows viral infections as also being responsible for significant morbidity and mortality in children [2].

Distinguishing between viral and bacterial infections remains a challenge, since the established bacterial detection methods, such as bacterial culture, can take a few days and even result in false negatives when the infection is located in non-accessible sites [3], or the sample is obtained after an antibiotic treatment [4]. Therefore, out of fear of not diagnosing and properly treating a potentially life-threatening bacterial infection, most clinicians decide to empirically administer antibiotics as a preventive tool while awaiting the bacterial culture test results [4,5]. Consequently, numerous viral infections are erroneously treated with antibiotics, contributing to the appearance of antibiotic-resistant bacteria [4,6]. Antibiotics have contributed to longer and healthier lives, but, as stated by the World Health Organization (WHO), their overuse, together with the absence of current-generation antimicrobial drugs, is enabling common infections and minor injuries to become fatal again.

The development of polymerase chain reaction (PCR)-based molecular assays has noticeably increased the capability to accurately diagnose old and emerging viral infections [7], and also the interrogation of multiple viruses in a single test [8]. Unfortunately, molecular assays have been less efficient in detecting bacterial infections, especially those caused by invasive infections [9]. Furthermore, because these tests point to the presence of nucleic acids, they might not identify the primary causative agent. Therefore, the detected pathogen could no longer be viable, and its presence may simply respond to a recent but unrelated illness [9], or even to an asymptomatic colonization.

In this context, the development of new diagnostic tools is one of the most important challenges of current public healthcare. They will play a central role in the fight against the emergence of bacterial resistance through precise and fast diagnosis, as well as facilitating the correct treatment of bacterial and viral infections.

The human transcriptome is a dynamic layer of information that changes according to cell types and organism conditions. Thus, host transcriptomics approaches not only hold the potential to shed light on the molecular pathogenesis of infectious diseases, but they may also enable the development of new diagnostic approaches based on the host gene expression response to specific pathogens [10,11]. Several host transcriptomic signatures in response to different infections were published in the last decade [4,12,13,14,15,16,17], but many of them were only focused on the specific pathogen and/or conditions studied, and usually in patients with the same age range or population background. As such, a multi-cohort analysis using publicly available data from different studies can help find common transcriptomic signatures, masking those expression patterns potentially related to specific pathogens, conditions, ages or genetic backgrounds, hence making the translation of these signatures to a generic test and its implementation in the clinical routine more straightforward [5,18,19,20].

In the present study, we explored host blood gene expression response to different infections to detect key transcriptomic changes related to viral or bacterial pathogens from a multi-cohort perspective. For this purpose, we downloaded 1209 transcriptomic sample profiles from public databases that correspond to 11 different gene expression studies from both microarray and RNA-seq data, containing bacteria- and virus-infected patients from different genetic population backgrounds and ages. We performed a multi-signature meta-analysis of the gene signatures that have been reported in these studies as potentially able to distinguish viral or bacterial infections. Through a machine learning approach, we were able to capture the best minimum transcriptomic signature among these gene candidates.

## 2. Results

To find the best candidates for a specific transcriptomic signature to distinguish viral from bacterial infections, we first combined the 11 different gene expression datasets including a total of 1209 samples (695 samples from viral infections and 514 samples from bacterial infections; Table 1; Table S1), obtaining 3025 common genes between them. Subsequently, we checked for the presence of the 163 different genes that have previously been published in these 11 studies as signature genes with the potential to differentiate between viral and bacterial conditions (Table S2) in the 3025 common genes (note that only a few of the 11 articles explored transcript signatures with the capability to separate groups of infection). As a result, 64 out of this initial list of 163 genes could be included in the meta-analysis gene set.

We performed an over-representation analysis with these 64 candidate genes (Table S2) using both Gene Ontology (GO) and Reactome as the reference pathway database. GO analysis pointed to an implication of these genes in immune response processes (*p*-adjusted: 3.24 × 10^–9^) mainly driven by the interferon I signaling pathway (1.26 × 10^–8^), the cytokine-mediated signaling pathway (*p*-adjusted: 2.23 × 10^–8^), neutrophil degranulation (*p*-adjusted: 1.34 × 10^–7^), innate immune response (*p*-adjusted: 2.58 × 10^–7^) and other biological processes related to mechanisms of defense against viral infection (*p*-adjusted: 9.68 × 10^–7)^ such as negative regulation of viral replication or cell cycle (Figure S1; Table S3). Similar results were achieved when carrying out the over-representation analysis with the Reactome database as the reference: interferon alpha/beta signaling (*p*-adjusted: 8.74 × 10^–9^), neutrophil degranulation (*p*-adjusted: 2.15 × 10^–6^) innate immune system (*p*-adjusted: 1.88 × 10^–4^) and cytokine signaling in the immune system (*p*-adjusted: 2.94 × 10^–6^) (Figure S2; Table S3). Some of the candidate genes are involved in the IL9 signaling pathway (statistically significative in both over-representation analyses; Table S3).

**Figure 1:**
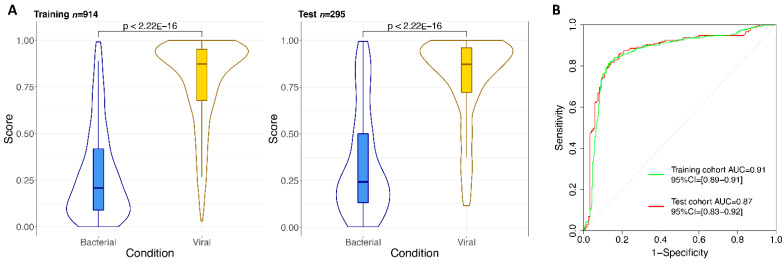
Signature performance based on the 14 genes in the training and test sets. (**A**) Violin and boxplots of the predicted values from the posterior mean. (**B**) Receiver operating characteristic (ROC) curves showing the area under the curve (AUC) and 95% confidence intervals (CIs).

Among these 64 candidate genes (Table S2), we searched for the minimum transcriptome signature that allows to discriminate between viral and bacterial infections using the optimal gene model size according to the Parallel Regularized Regression Model Search (PReMS) algorithm. To study the expression patterns of these candidate genes in our multi-cohort database, we followed a cross-validation strategy that randomly divides the whole dataset into a training (75% of the samples) and a test set (remaining 25% of the samples) both including bacteria- and virus-infected samples. First, we carried out an exploratory analysis on the training set using all candidate genes in the model to assess how the predictive log-likelihood changes with the number of genes included in the signature (Figure S3a). We found that the optimal model was composed of 14 genes (Figure S3b) that clearly separate viral from bacterial infections (Figure 1A) in both the training and the test set (*p*-value <2.22 × 10^–16^). We also computed the area under the curve (AUC) of the 14-transcript signature in the training and test cohorts, obtaining values of 0.91 (95%CI: 0.89–0.91) for the training cohort and 0.87 (95%CI: 0.83–0.92) for the test cohort (Figure 1B).

**Figure 2:**
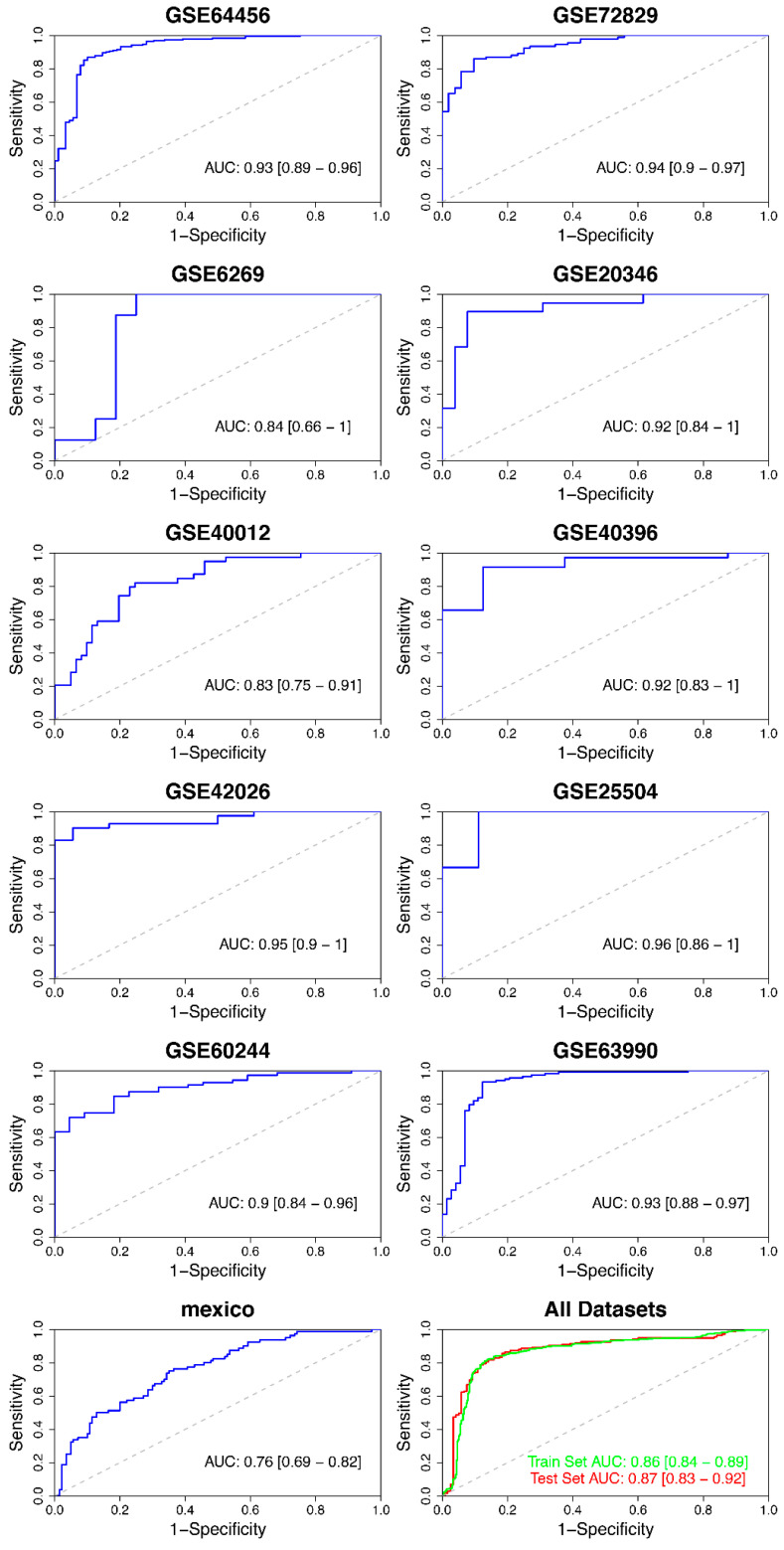
Evaluation of the 3-transcript signature performance in individual studies as well as in the training and test cohorts. AUC values and 95% CI are provided.

We analyzed in more detail the predictive log-likelihood (Figure S3b) calculated from the training cohort after applying the machine learning algorithm to strike a balance between the size and the accuracy of the gene expression signature. We found that the minimum signature of three genes keeps a predictive value that is only slightly lower compared with the 14-transcript signature; in other words, the addition of genes to the three-transcript model adds very little to the overall predictive value. The minimal signature is composed of genes *BATF* (Basic Leucine Zipper ATF-Like Transcription Factor), *ISG15* (ISG15 Ubiquitin Like Modifier) and *DNMT1* (DNA Methyltransferase 1). This signature differentiated bacterial from viral infections with high accuracy (Figure S4), reporting an AUC value of 0.86 (95%CI: 0.84–0.89), with a sensitivity of 0.81 and a specificity of 0.87 (Table 2; Figure 2) in the training set. The performance was equivalent in the test cohort, with an AUC of 0.87 (95%CI: 0.83–0.92), a sensitivity of 0.82 and a specificity of 0.86 (Table 2; Figure 2).

We further evaluated the performance of the 3-transcript model to differentiate viral from bacterial cases in each individual study; AUC values calculated ranged from 0.76 to 0.96 (Table 2, Figure 2). The lower value of AUC (AUC: 0.76 (95%CI: 0.69–0.82); sensitivity: 0.75 and specificity: 0.65) was achieved in the Mexican cohort (RNA-seq data; GSE69529), and this low value probably reflects the heterogeneous nature of the cohort, which included patients affected by a mild disease.

## 3. Discussion

Both viral and bacterial infections occur with unspecific clinical symptoms, especially in early stages of the disease. In fact, viral and bacterial infections are often indistinguishable when considering only clinical settings and, therefore, empirical therapies are often administered as a preventive measure. The excessive use of antibiotics has led to an alarming increase in bacterial resistance and, in parallel, healthcare costs. The first step towards more precise antibiotic administration is the availability of faster, more sensitive, and accurate diagnostic tests. However, the tests currently available have several limitations; for instance, the gold standard of using bacterial cultures usually takes a long time to produce results. Although microbiological diagnosis has improved since the emergence of PCR-based assays, these tests do not always detect the causative pathogen, as available panels only interrogate the most frequent pathogens (requiring a priori suspicion of the pathogen), and sometimes they detect residual remains of a past infection.

In the present study, we conducted a multi-cohort meta-analysis using high-throughput (microarray and RNAseq) data available in public databases (*n* = 1209 samples) from blood transcriptomic studies including virus and bacteria-infected patients to find the best minimum gene expression signature that differentiates between both types of infections in all possible scenarios. Meta-analysis of transcriptomic data has proven to be a useful approach to discover gene expression signatures specific to different infectious diseases [5,18,20], raising the statistical power compared with individual studies, and finding common trends in transcriptomic response under different conditions, pathogens, and demographic features. Using a gene signature candidate approach following a PReMS algorithm, we obtained a biosignature of 3-gene transcriptomics that accurately distinguishes viral from bacterial infections with high sensitivity and specificity. This signature also performed well when validated in all individual studies (Table 3; Figure 2), pointing to the functional versatility of the three-transcript signature in very different infection contexts. Two of the three genes in the signature, namely *BAFT* and *ISG15*, are both related to immune processes and, while the former is involved in several differentiation processes of some immune cells, the latter plays a key role in the immune response to RNA and DNA viruses [30,31,32]. On the other hand, the *DNMT1* gene encodes for a protein that is responsible for maintaining DNA methylation patterns after replication and it has been shown that some viral [33,34] and bacterial [35] infections can induce the expression of this gene.

Although knowledge of the functional features of these genes is of great interest, the most important issue in the context of biomarker discovery research is their capability to differentiate both types of infections, regardless of their role in the context of the pathophysiology of the disease. It occurs very often that candidate genes have unknown function, but this fact does not invalidate its potential to have specific diagnostic biomarkers. For instance, Herberg et al. [4] discovered a two-transcript signature from microarray expression data, which discriminated between viral and bacterial infections with no known function of the genes involved. Despite this, the two-transcript signature was successfully tested and validated in prospective and other retrospective cohorts, and using different gene-expression technologies [5,6,36]. In the same line, two long non-coding RNAs have been recently proposed as biomarkers associated with viral infections, showing high performance capability in separating viral from healthy phenotypes [36]; their role, however, is completely unknown.

The main advantages of a 3-gene signature are its easy implementation in a diagnostic test, given the low number of genes needed, and its functionality under different conditions derived from the multi-cohort study. Even though RNA-seq and microarrays are emerging as the most powerful screening approaches to discover host RNA signatures related to infectious diseases, both have inherent problems such as a higher error rate than traditional Sanger sequencing, standardization, and reproducibility issues [10]. Therefore, before any biomarker is translated into a clinical test, it needs to be validated using well-standardized technologies [6] in proper clinical settings. Consequently, further effort is needed to validate the three-biomarker signature using robust molecular techniques such as real time-PCR (qPCR) [6] or nCounter (Nanostring^®^) [10]. The qPCR is currently the “gold standard” in gene expression studies. Many studies have proven that qPCR is a suitable method to validate microarrays and RNA-seq findings, reporting a strong correlation between microarray and qPCR results [37]. Furthermore, qPCR-based assays are already widely used in hospital settings because this is a technique with high accuracy, which is also relatively cheap and fast [6]. However, establishing a detailed laboratory qPCR protocol that includes a careful selection of reference genes for each specific condition and good laboratory practices is crucial to successfully convert a host transcriptional signature into a qPCR assay that can be used in a diagnostics laboratory routinely [6].

Even though the development of a bedside test based on host transcriptomic biomarkers is highly desirable, this goal is not easy to achieve due to technical limitations. Nonetheless, this situation will most probably change soon thanks to new emergent technologies that will allow for sensitive and qualitative detection of gene expression within a short time frame. It is likely that in the next few years, we will see the application of the first host gene expression diagnostic tests for infectious diseases in clinical settings and, more importantly, an improvement in the diagnosis and treatment of infectious diseases [10].

## 4. Conclusions

Our results suggest that different infectious diseases are associated with different patterns of genes that turn on or off, constituting specific molecular signatures, which can be used to quickly identify viral or bacterial infections. We found three genes, namely *BATF*, *ISG15* and *DNMT1*, which can distinguish viral from bacterial infections in a wide range of cohorts including different pathogens, ages and populations, and with potential to become clinical biomarkers for infectious diseases in a clinical setting. As occurred in previous studies [4,5,6,15,36], the role of biomarkers of infection is often unknown; this fact, however, does not diminish the importance of their capability to distinguish viral from bacterial infections. In our study, the concurrence of these biomarkers in a significant number of independent studies points to their important role in the process of infection, and this observation strongly suggests the need for further investigations.

The present study represents a step forward towards the use of host gene expression signatures in clinical settings. Due to the nature of our meta-analysis that uses retrospective data from 11 previously published studies, a validation cannot be done using the original samples. Therefore, further effort will be needed to collect new samples from viral and bacterial infected patients to further explore the 3-transcript signature in a new prospective cohort. Moreover, the translation of the selected transcriptomic biomarkers into a clinical test for diagnosis, prognosis or risk assessment needs further validation, as well as consideration of different scenarios, including illness severity, time points in the course of the infectious disease, parasitic infections, and other inflammatory diseases. In this context, a 3-transcript qPCR validation assay or alike (e.g., using the Nano String platform) might be also of interest before developing a point-of-care test.

There are still many challenges to overcome before host gene expression signatures can be introduced into a point-of-care molecular diagnostic test. However, signatures based on host gene expression biomarkers have a great potential for the diagnosis of infectious diseases; we envisage that their use in clinical diagnostic tests will skyrocket in the next few years.

## 5. Material and Methods

### 5.1. Sample Groups

We queried the public gene expression microarray repository Gene Expression Omnibus (GEO) for human gene expression datasets using the following terms: “viral” and/or “bacterial”. We retained only those studies containing microarray expression or RNA-seq data from whole blood samples of virus- or bacteria-infected patients. Eleven studies (*n* = 1209 samples) were included in the metanalysis (see details in Table 1): GSE64456 [19] (*n* = 279), GSE72829 [4] (*n* = 144), GSE6269 [22] (*n* = 24), GSE20346 [23] (*n* = 45), GSE40012 [24] (*n* = 100), GSE40396 [25] (*n* = 43), GSE42026 [26] (*n* = 59), GSE25504 [27] (*n* = 12), GSE60244 [28] (*n* = 93), GSE69529 [21] (*n* = 220) and GSE63990 [29] (*n* = 190), including patients with bacterial and viral infections (Table S1).

### 5.2. Data Processing and Statistical Analysis

To merge and integrate the public viral vs. bacterial transcriptomic studies, we first normalized and pre-processed each dataset separately using the package *Lumi* [38] for Illumina^®^ microarrays data and the package *Oligo* [39] for Affymetrix^®^ datasets. RNA-seq data were pre-processed as described in [5].

We first merged these databases keeping only common genes included in all of them. Subsequently, we used the R package COCONUT (COmbat CO-Normalization Using conTrols) to combine all datasets into one and reduce batch effects in the meta-analysis [20]. After that, we only used for the follow-up analyses the candidate biomarkers reported in these studies as capable of differentiating between viral and bacterial infections. Only 64 out 163 candidate genes were present in all databases (Table S2) and, therefore, these 64 candidate genes were used as input to explore the minimum specific transcript signature for distinguishing viral from bacterial infection. We used PReMS [40] in a randomly split dataset removing healthy controls: training set (*n* = 914) and validation set (*n* = 295). PReMS investigates different logistic regression models built from optimal subsets of the candidate genes while increasing the model size iteratively. PReMS was the preferred method as it tends to choose signatures with a smaller number of genes without losing model accuracy, which would facilitate its future translation into a point-of-care test [10]. We tested first a model with a maximum of 15 genes and then explored how the predictive log-likelihood values change with the number of genes to find the signature with the minimum number of transcripts with optimum performance and facilitate its translation into the clinical routine.

Finally, the accuracy of the model estimated by PReMS was calculated as the AUC using the R package *pROC* [41] in both training and test cohorts as well as in all independent studies from the multi-cohort study. The Wilcoxon test was used to assess statistical significance between viral and bacterial groups. Functional pathways analysis was carried out through the *Clusterprofiler* [42] R package. We used the package *enrichplot* [43] for graphically displaying the results obtained. Heatmap representation of the top 14 genes from the optimal model was carried out with the ComplexHeatmap R package [44].

All analyses and graphical representations were conducted using R software version 3.6.4 (www.r-project.org/, accessed on 26 January 2021).

## Figures and Tables

**Table 1:** Samples included in the meta-analysis (GEO: Gene Expression Omnibus). Platform: lllumina (I), Affymetrix (A); MA: microarray; Cohort: children (C); Adult (A); Source: whole blood (WB), peripheral blood mononuclear cell (PBMCs).

GEO ID	*n* (Virus)	*n* (Bacteria)	Platform Description	Cohort	Source	Reference
GSE69529	80	140	HiSeq 2500 (I); RNA-seq	C	WB	[21]
GSE64456	190	89	HT12 V4 (I); MA	C	WB	[19]
GSE72829	92	52	HT12 V3 (I); MA	C	WB	[4]
GSE6269	8	16	HG U133A Array (A); MA	C	PBMCs	[22]
GSE20346	19	26	HT-12 V3 (I); MA	A	WB	[23]
GSE40012	39	61	HT-12 V3 (I); MA	A	WB	[24]
GSE40396	35	8	HT-12 V4 (I); MA	C	WB	[25]
GSE42026	41	18	HT-12 V3 (I); MA	C	WB	[26]
GSE25504	3	9	HG U133 Plus 2.0 Array (A); MA	C	WB	[27]
GSE60244	71	22	HT-12 V4 (I); MA	A	WB	[28]
GSE63990	117	73	HG U133 Plus 2.0 Array (I); MA	A/C	WB	[29]
Totals	695	514				

**Table 2:** AUC, sensitivity and specificity of the 3-transcript signature.

Study	Thresholds	Sensitivity	Specificity	AUC	95% CI
GSE64456	10.80	0.87	0.90	0.93	0.89–0.96
GSE72829	2.96	0.86	0.90	0.94	0.90–0.97
GSE6269	12.49	1.00	0.75	0.84	0.66–1.00
GSE20346	7.00	0.89	0.92	0.92	0.84–1.00
GSE40012	7.07	0.82	0.75	0.83	0.75–0.91
GSE40396	11.64	0.90	0.88	0.92	0.83–1.00
GSE42026	8.27	1.00	0.94	0.95	0.90–1.00
GSE25504	10.34	1.00	0.89	0.96	0.86–1.00
GSE60244	9.75	0.72	0.95	0.90	0.84–0.96
GSE63990	6.83	0.93	0.88	0.93	0.88–0.97
GSE69529	792.62	0.75	0.65	0.76	0.69–0.82
Training set	439.56	0.81	0.87	0.86	0.84–0.89
Test set	439.77	0.82	0.86	0.87	0.83–0.92

**Table 3:** Genes included in the viral vs bacterial 3-gene transcriptomic signature. LRC = logistic regression coefficient.

Gene Symbol	Gene Name	LRC
*BATF*	Basic Leucine Zipper ATF-Like Transcription Factor	−1.16
*ISG15*	ISG15 Ubiquitin Like Modifier	0.64
*DNMT1*	DNA Methyltransferase 1	1.24

## Data Availability

Not applicable.

## Supplementary Materials

The following are available online at https://www.mdpi.com/1422-0067/22/6/3148/s1.
