# Supplementary figures and images for: Identification of a Minimal 3-Transcript Signature to Differentiate Viral from Bacterial Infection from Best Genome-Wide Host RNA Biomarkers: A Multi-Cohort Analysis
Source: Int J Mol Sci. 2021 Mar 19;22(6):3148. doi: 10.3390/ijms22063148 (PMC8003556; doi:10.3390/ijms22063148)

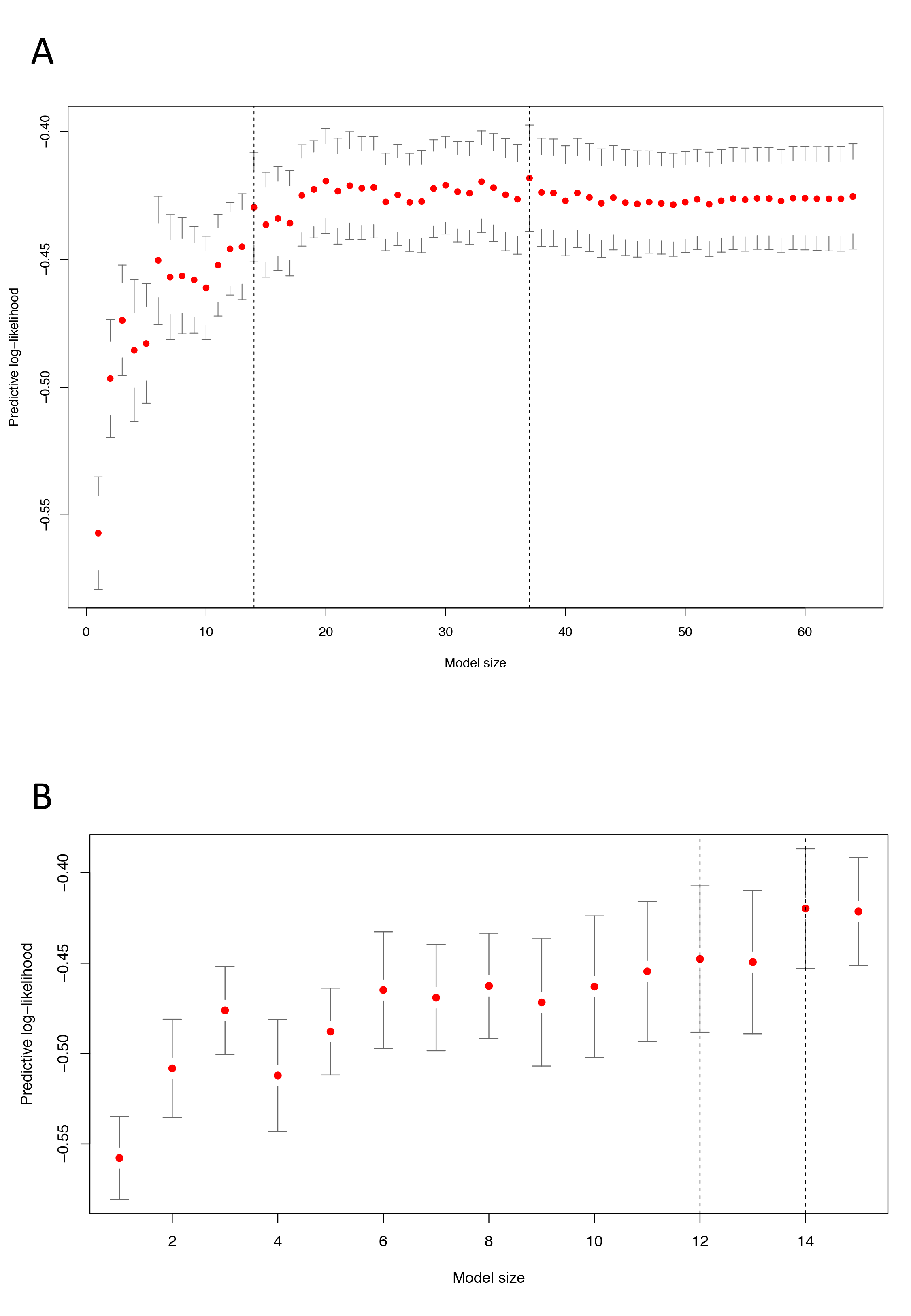

Supplement: Supplementary file 1 [file ijms-22-03148-s001.zip › ijms-1106436-sup/Figure S3.tif]

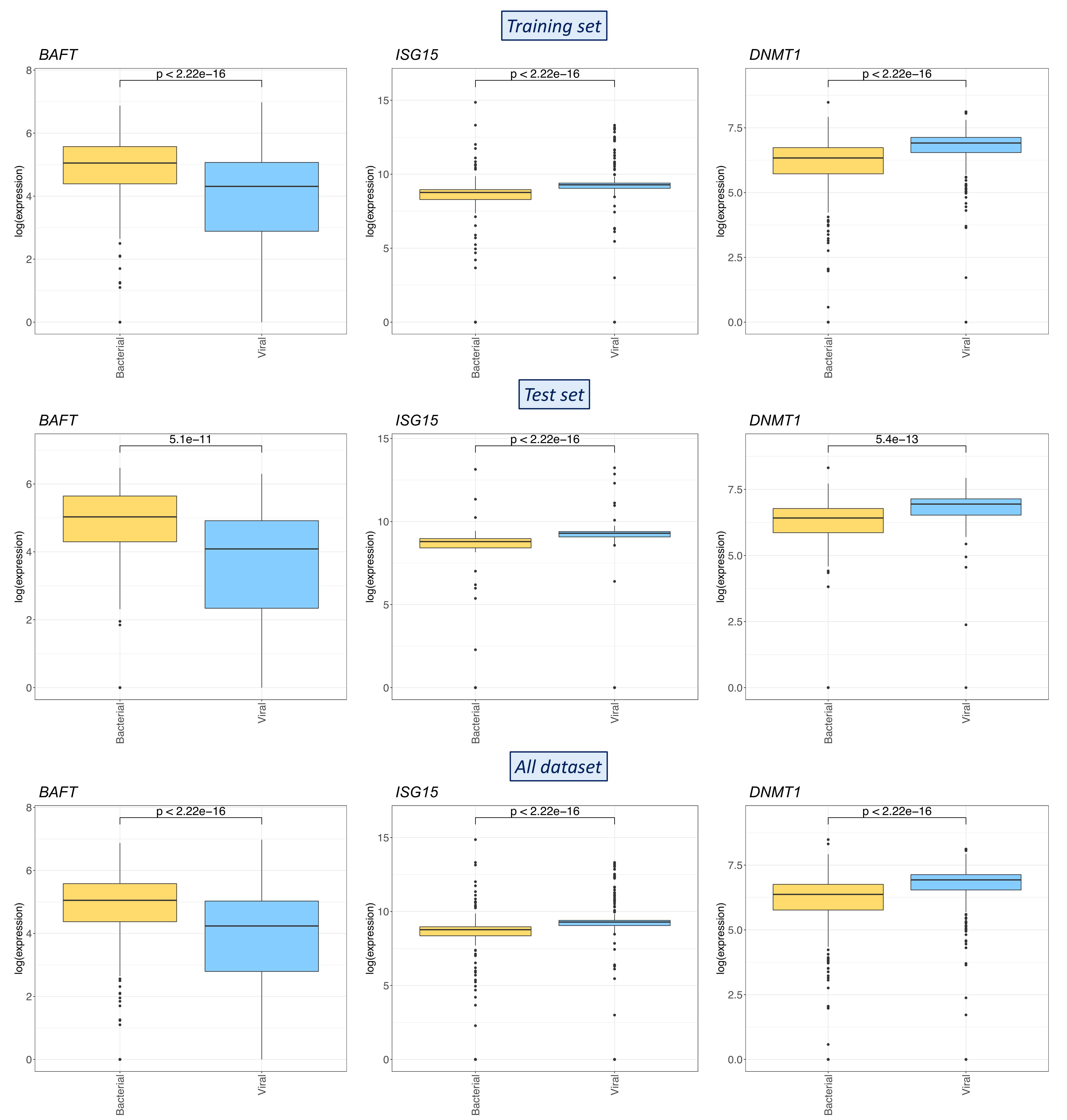

Supplement: Supplementary file 1 [file ijms-22-03148-s001.zip › ijms-1106436-sup/Figure S4.tiff]

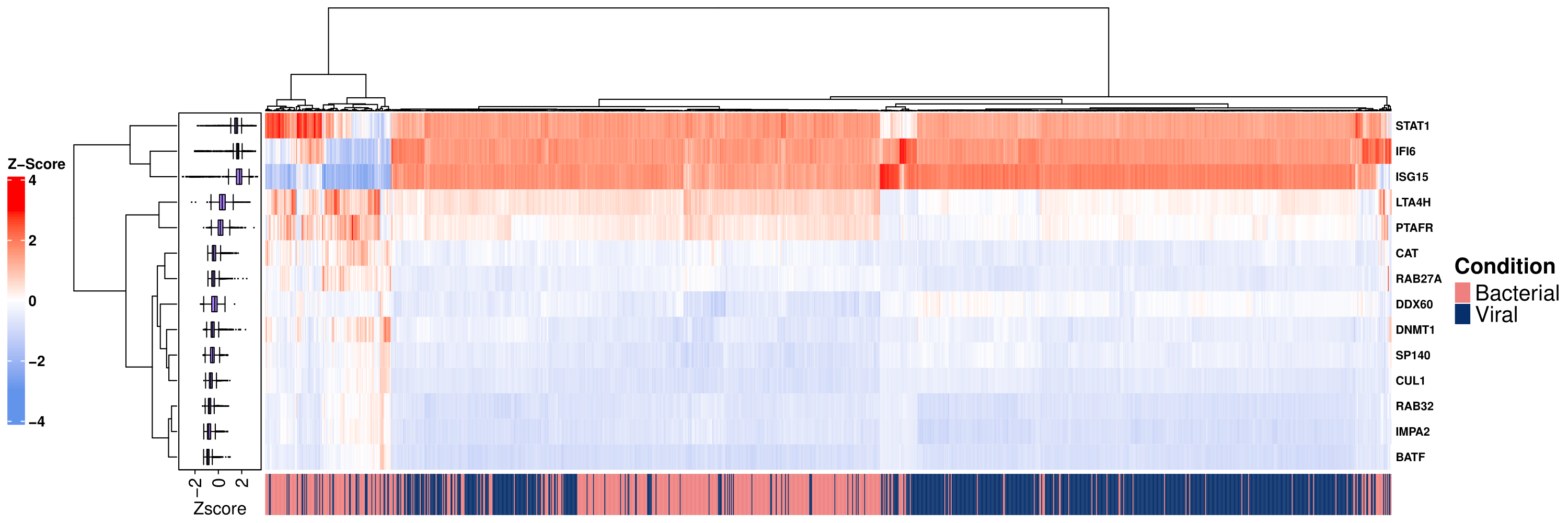

Supplement: Supplementary file 1 [file ijms-22-03148-s001.zip › ijms-1106436-sup/Figure S5.tiff]
